# Innovative Diagnostic Methods for Early Prostate Cancer Detection through Urine Analysis: A Review

**DOI:** 10.3390/cancers10040123

**Published:** 2018-04-18

**Authors:** Carmen Bax, Gianluigi Taverna, Lidia Eusebio, Selena Sironi, Fabio Grizzi, Giorgio Guazzoni, Laura Capelli

**Affiliations:** 1 Politecnico di Milano, Department of Chemistry, Materials and Chemical Engineering “Giulio Natta”, Piazza Leonardo da Vinci 32, 20133 Milan, Italy; lidia.eusebio@polimi.it (L.E.); selena.sironi@polimi.it (S.S.); laura.capelli@polimi.it (L.C.); 2 Humanitas Clinical and Research Center, Department of Urology, via Manzoni 56, Rozzano, 20089 Milan, Italy; gianluigi.taverna@humanitas.it (G.T.); giorgio.guazzoni@humanitas.it (G.G.); 3 Humanitas Clinical and Research Center, Department of Immunology and Inflammation, via Manzoni 56, Rozzano, 20089 Milan, Italy; fabio.grizzi@humanitasresearch.it

**Keywords:** prostate cancer diagnosis, biomarkers, electronic nose, olfaction, gas chromatography, VOCs, trained dogs

## Abstract

Prostate cancer is the second most common cause of cancer death among men. It is an asymptomatic and slow growing tumour, which starts occurring in young men, but can be detected only around the age of 40–50. Although its long latency period and potential curability make prostate cancer a perfect candidate for screening programs, the current procedure lacks in specificity. Researchers are rising to the challenge of developing innovative tools able of detecting the disease during its early stage that is the most curable. In recent years, the interest in characterisation of biological fluids aimed at the identification of tumour-specific compounds has increased significantly, since cell neoplastic transformation causes metabolic alterations leading to volatile organic compounds release. In the scientific literature, different approaches have been proposed. Many studies focus on the identification of a cancer-characteristic “odour fingerprint” emanated from biological samples through the application of sensorial or senso-instrumental analyses, others suggest a chemical characterisation of biological fluids with the aim of identifying prostate cancer (PCa)-specific biomarkers. This paper focuses on the review of literary studies in the field of prostate cancer diagnosis, in order to provide an overview of innovative methods based on the analysis of urine, thereby comparing them with the traditional diagnostic procedures.

## 1. Introduction

Prostate cancer (PCa) is the most common diagnosed cancer in Europe and America and the second most common cause of cancer death among men [1]. The National Cancer Institute estimates 161,360 new cases of PCa and 26,730 deaths in 2017 [2].

PCa is an asymptomatic tumour, which starts occurring in men aging 20–30 years, but can be detected only in the fourth-fifth decade [3]. Indeed, symptoms appear only when the disease has reached an advanced stage, reducing the number of adoptable treatments and patients’ chances of surviving [4].

The long latency period of PCa and its potential curability in early stages make this disease a perfect candidate for screening programs [5]. Nevertheless, PCa diagnosis is challenging because of the late onset of symptoms and the limits of the current diagnostic procedures.

Serum prostate specific antigen (PSA) level and digital rectal examination (DRE) constitute the major screening tests for PCa diagnosis, while the transrectal ultrasound-guided prostate biopsy (PBs) provides the final confirmation of cancer presence [6]. A PSA serum level higher than 4 ng/mL, currently adopted as marker, is not a sure sign of PCa, because it may reflect also inflammatory diseases (e.g., prostatitis, irritations), non-cancer related benign prostatic hypertrophy (BPH) or diet alterations, resulting in false positives [5]. Moreover, PSA test does not allow to differentiate aggressive PCa from indolent diseases [7]. Therefore, a PBs is necessary for a final diagnosis [7]. However, PBs may entails risks for patients (i.e., bleeding or subsequent infections) [8] and offers low levels of accuracy (i.e., only 30% detection rate at first biopsy), especially when the tumour is small [9]. Therefore, suspected patients should undergo repeated PBs in order to improve the detection rate. The inefficacy of PCa screening programs result in patients’ overtreatment [6], and consequent increase of health spending [10].

In order to improve the accuracy of the diagnosis, researchers are rising to the challenge of developing innovative diagnostic tools able of identifying PCa during its early stage that is the most curable [11]. Effective screening tests should be non-invasive, easily accessible, quickly quantifiable, reliable, and reproducible [12,13]. They should have high sensitivity, specificity, low financial burden on patients, and the lowest possible risk level [14]. An improvement of the design of screening programs requires a better characterisation of the disease biology and the investigation of disease dynamics and molecular diagnostics, which determines whether the cancer will be aggressive or indolent, in order to avoid overtreatment [14]. 

Recent years have seen remarkable progresses in the characterisation and the quantification of biological molecules and the explanation of their roles in cells, opening the possibility to identify molecular changes and metabolic pathways specific for cancer [7]. 

Since metabolic alterations, typical of cell neoplastic transformation processes, lead to peroxidation of membrane components and consequent release of volatile organic compounds (VOCs) [15], the investigation of biological fluids can provide useful information about pathological conditions [7]. Therefore, the interest in their characterisation aimed at the identification of tumour-specific compounds has increased significantly.

In the scientific literature, different approaches have been proposed. Many literary studies [16,17,18,19,20,21,22,23,24] focused on the identification of a cancer-characteristic “odour fingerprint” emanated from biological samples, instead of a detailed chemical mapping, through the application of sensorial or senso-instrumental analyses. Other researchers [25,26,27,28,29,30,31,32,33,34,35,36,37,38,39,40] suggested a chemical characterisation of biological fluids or of their gaseous headspace with the aim of identifying PCa-specific biomarkers.

Commonly, blood, urine, prostatic or seminal fluid, and prostate tissue, are analysed for these purposes.

Blood is rich in human proteins, which are considered potential biomarkers for PCa detection and prognosis. However, the masking effects of high-abundance compounds worsen the identification of low-abundance proteins, because of the wide range of concentrations and the high amounts of interfering compounds [41].

Prostate tissue is the best source of PCa specific biomarkers, but it is the most invasive, expensive, and risky of sampling sites, entailing side effects such as bleeding or infections [6].

Semen provides information directly from the prostate, but it is characterised by high variability, and it may be difficult to collect it in men with other diseases [7].

Urine has been investigated for centuries as source of useful information for the assessment of diseases [42,43,44,45]. Because urine is the carrier of blood wastes, it may provide information not only from kidney and urinary tracts, but also from distant organs via plasma obtained through glomerular filtration [6]. Compared to other biological fluids, urine has the advantages of being inexpensive, rich in metabolites, easy to handle, and available in large amounts, without requiring invasive treatments for collection [46,47,48,49]. However, it is characterised by very low concentrations of possible biomarkers and high variability among patients depending on gender, age, hormonal status, diet, or physical activity [50,51,52,53].

This paper focuses on the review of literary studies in the field of medical diagnostics about innovative methods for early PCa detection based on the analysis of urine, considering a timeframe of publication between 2008 and 2017. In particular, this review aims to provide an overview of innovative methods for PCa diagnosis, thereby comparing them with the traditional diagnostic procedures.

The following sections provide a general overview of traditional diagnostic procedures (Section 2) and of the state-of-the-art of alternatives proposed in recent years (Section 3). Innovative techniques considered are grouped by type of study, according to the method adopted: sensorial methods involving the use of high-trained dogs (Section 3.1.1), senso-instrumental methods based on the adoption of electronic noses (Section 3.1.1), and analytical techniques based on the chemical characterisation of urine (Section 3.2). 

Given the huge number of possible techniques for the chemical characterisation of urine samples [54], in this review, only approaches based on liquid or gas chromatography and mass spectrometry were considered. These analytic techniques are the most consolidated and allow detecting metabolites at very low concentrations with high sensitivity [54], although recent studies based on magnetic resonance seem to be very promising, especially for the analysis of prostatic fluid or tissue [55,56,57].

## 2. Overview of Traditional Diagnostic Methods

This section aims to give a general overview of the traditional diagnostic procedure for PCa detection. The detailed description of the current diagnostic methods falls out of the scope of this paper, besides, it is already object of other exhaustive reviews, papers, and books [58,59,60,61,62]; for this reason, this section is limited to highlight the main issues of current diagnostics for further comparison with the investigated innovative techniques.

Current screening for PCa is based on the measurement of prostate specific antigen (PSA) serum level. The PSA is a 33 kDa serine protease responsible for the controlled release of sperm, which is secreted into the fluid of the glandular ducts [3,63]. In a normal prostate, only small amounts (i.e., <4 ng/mL) of PSA reach the circulation system by leaking backwards into extracellular fluid and diffusing into circulation [3]. Conversely, in case of PCa, PSA serum level is higher, because of the derangement of epithelial cells architecture and polarisation, which causes the loss of normal secretory pathways into the prostatic ducts [3]. 

Literature studies about PCa development [64,65] demonstrated that the concentration of serum PSA and its doubling time or PSA velocity reflect the grow rate of PCa. However, PSA test is characterised by low diagnostic accuracy (i.e., specificity around 33% and sensitivity around 86%) [9]. Indeed, higher PSA serum levels may be associated with other non-cancer related diseases, such as prostatitis, irritations, benign prostatic hypertrophy (BHP), or also diet alterations [5]. Moreover, PSA falsely diagnoses indolent PCa, which tends to grow and spread slowly. Thus, the lack of diagnostic accuracy associated with this method often results in patients’ overtreatment, and increase of health spending [66,67]. Approximately 10% of men over 50 years old have a PSA serum levels over 4 ng/mL, but among them, only 30% have a PCa confirmed by further investigations [3]. Wilt et al. [68] proved that, after positive PSA test, patients who underwent radical prostatectomy did not have a significant reduction in mortality with respect to those who opted for active surveillance over a 12 year follow up.

Since PSA occurs in serum in various molecular forms (i.e., free PSA or complexes with inhibitors), many efforts have been made to increase the diagnostic accuracy of PSA test through the quantification of these different forms [69,70,71]. Although the consideration of different PSA molecular forms (ratio free to total PSA, isoforms p2PSA) increases cancer detection rates [72,73], they are not useful for the diagnosis of prostate cancer by itself [74].

Therefore, in case of high PSA serum level, the patient usually undergoes digital rectal examination (DRE). As most cancers are located in the peripheral posteriori area, DRE may detect bumps, soft or hard spots, or abnormal masses [75]. However, DRE is not capable of detecting early PCa, because a minimum tumour volume of 0.2 mL is needed for a possible diagnosis: DRE will detect a cancer only in the 0.1–4% of asymptomatic men considered [75]. Patients having a normal DRE outcome, though PSA serum level is markedly altered, will watchfully wait, repeat PSA and DRE periodically, and undergo further invasive tests (i.e., the biopsy). 

**Figure 1:**
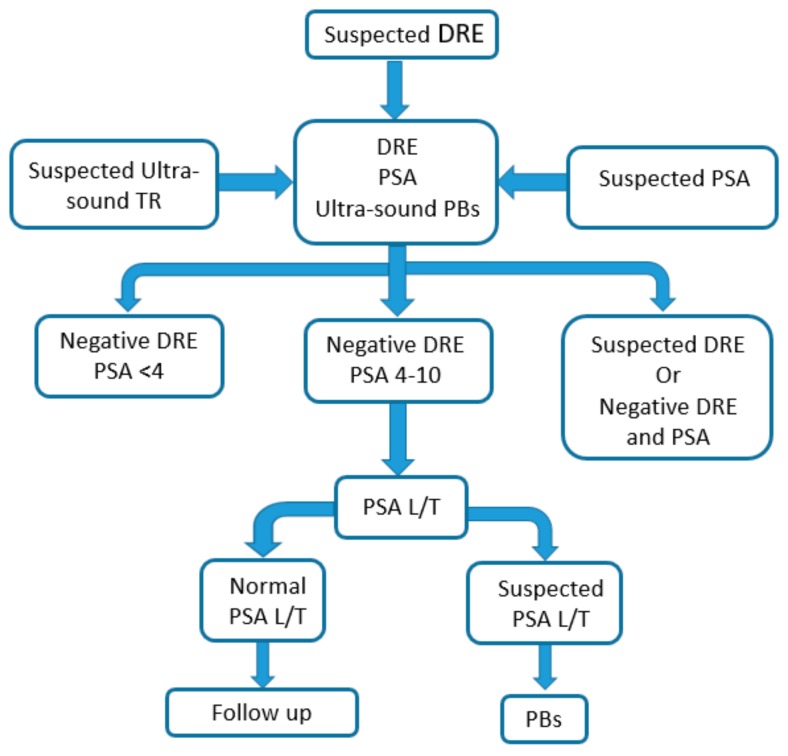
Prostate cancer current diagnostic procedure. (DRE, digital rectal examination; PSA, prostate specific antigen; PBs, transrectal ultrasound-guided prostate biopsy)

Transrectal ultrasound-guided prostate biopsies (PBs) provide the confirmation of PCa presence. This exam is invasive, expensive, and risky. It may cause subsequent infections, erectile dysfunction, and urinary incontinence [76]. Many studies have demonstrated that there is a significant increase of the hospitalisation rate due to infections following biopsy [8,77]. Moreover, the overall cancer detection rate is 30% at first biopsy [19,78,79]. Therefore, in general, repeated biopsies are performed to improve cancer detection rate. Although the use of MRI has led to the implementation of new diagnostic strategies, including the development of equipment that facilitates MRI/TRUS fusion-guided biopsies, and consequently, the increase of the positive biopsy detection rate, there remains a need for standardisation, technical improvements, and appropriate training of radiologists to guarantee sufficient quality and reproducibility [80]. The current PCa diagnostic procedure is summarised in Figure 1.

Considering the limits of the actual diagnostic procedure for PCa detection, the need of a more accurate prognostic method able of reducing overtreatment of low risk patients, unnecessary biopsies and radical prostatectomies is evident.

## 3. Innovative Techniques

Given the abovementioned issues related to the current diagnostic procedures for PCa, in recent years, many researches [19,69,81] have focused on the development of more accurate diagnostic tests compared to traditional ones. The main goal of this research field is the identification of cancer during its early stage, in order to decrease mortality and treatment costs related to PCa. For this purpose, new tests should be capable of providing accurate information about the prognosis and the most adequate treatment plan to be adopted.

This review aims to provide an overview of the state-of-the-art of innovative diagnostic techniques for early PCa detection based on urine analysis. The in-depth analysis of literature highlighted that this research field is still in progress, and different approaches have been proposed as alternatives of the current diagnostic procedure. Considering the differences between those approaches, for clarity of exposure, we decided to group the considered literary works by the type of analysis proposed, which are

- sensorial analysis, which relies on the mammalian sense of smell;
- senso-instrumental analysis, which tries to gather information about the olfactory properties of the analysed sample (urine) by means of specific instruments (i.e., electronic noses);
- chemical analysis, which relies on analytical techniques for the identification and quantification of chemical compounds (e.g., GC-MS).

Sensorial and senso-instrumental analyses have, in common, the characterisation of the odour emanated from biological samples aimed to the identification of a cancer-characteristic “odour fingerprint”, thus considering the olfactory properties of urine as a whole [82,83,84,85]. As already mentioned, sensorial methods are based on the direct characterisation of odours relying on the general human or animal sense of smell. In the particular field of diagnostics, some researchers [15,86] proved the capability of highly trained dogs to detect alterations of body odours associated with specific illnesses.

Senso-instrumental methods are based on instruments that mimic the mammalian olfactory system. Those instruments, commonly named electronic noses (EN), provide a global characterisation of the odorous mixture. EN typically comprise an array of electronic chemical sensors with partial specificity, and an appropriate pattern recognition system capable of recognising simple or complex odours [87].

Conversely, chemical analyses are based on the chemical characterisation of liquid urine or its headspace aiming to the detection of PCa biomarkers and the quantification of their amounts. They provide a detailed chemical mapping of urine through the adoption of different analytic techniques, such as gas or liquid chromatography coupled with mass spectrometry, solid phase microextraction or ion exchange liquid chromatography [27,30,36].

### 3.1. Olfactory Fingerprint Investigation

It has long been known that VOCs emanating from biological fluids contain information about the internal biochemistry of the human body. In ancient medicine, the diagnosis of different diseases relied on the sensorial analysis of biological fluids. Hippocrates [88] attributed all diseases to disorders of fluids, and proposed a diagnostic protocol that included observing skin and urine colour or urine tasting. 

In recent years, the diagnostic usefulness of biological fluid has been revaluated. Indeed, several research studies [86,89,90] have been published regarding the investigation of biological fluid odours with the aim of identifying the presence of VOCs that are specifically related to different diseases. In the field of PCa diagnosis, some research groups proved the capability of highly trained dogs to detect alterations of body odours associated with cancer, while others tried to develop instrumental methods, proposing ENs as diagnostic tools.

#### 3.1.1. Trained Dogs

Dogs are widely employed by police for detecting explosives and drugs and locating missing persons [91,92]. Dogs’ olfactory system is capable of detecting odours as low as part per trillion [93], thanks to characteristic anatomic factors, such as the increased dimension of their olfactory epithelium, the huge number of olfactory receptors and the dense innervations of their olfactory mucosa [94].

The first trial investigating the feasibility of dogs’ adoption for early PCa detection through the analysis of urine samples was published by Gordon et al. [16] in 2008. They trained four dogs of different breeds to discriminate men affected by PCa from healthy volunteers by the clicker training method. The training was performed by dogs’ owners, who followed the same general outline for training protocol. During the training phase, cancerous urines were progressively presented to dogs against empty test tubes, water, diluted control urines, and finally, full-strength control urines in order to progressively complicate the system. In the latter training phase, dogs were presented with one positive urine against six control samples. Then, dogs’ ability was tested in 33 blind runs. Each run contained six control samples and one cancerous urine placed in screw-top vial in randomised order. Dogs’ performance in blind runs was worse than expected. Sensitivity achieved was around 20% for all dogs involved, while specificity was higher than 60% only for two dogs. This outcome may be explained, as suggested by the authors themselves, considering protocol deficiencies of training, i.e., absence of professional team of trainers, of a central training site and of a standardised procedure.

Cornu et al. [17] tested the ability of a Belgian Malinois shepherd to discriminate PCa and control urines. The dog was trained by the clicker training method for 16 months. Then, it was involved in a double-blind procedure that consisted of consecutive runs. For each run, the dog was presented five controls and one cancer anonymised sample. For the analyses, urine samples, stored at −4 °C, were slowly heated to 37 °C. The dog had to scent, successively, the six samples, and after a mean time of 30 s, it had to sit in front of the cancer sample. In case of success, the sample was classified as true positive; otherwise, it was considered as a false positive. Their study involved 59 men affected by PCa and 49 control subjects, and no exclusion criteria regarding medical history, diet, drugs, or tobacco consumption was considered in the selection of participants. All patients were classified as controls or PCa after undergoing PBs. The descriptive analysis for dogs’ performance evaluation was performed with XLStat for Windows (Addinsoft, Paris, France) and the sensitivity and specificity achieved were of 91%.

Elliker et al. [18] attempted to train ten dogs of seven different breeds for the PCa detection adopting a two-stage training procedure. During the first stage of training, dogs learnt to find and indicate PCa samples, while during the second phase, they became able to discriminate PCa samples from controls. Urine samples were frozen at −20 °C within 10 min after collection, and defrosted in a 37 °C water bath before dogs’ examination. Following training, three double-blind tests were performed for the two dogs that gave the best performances. During the blind test, the dogs were presented with 15 arrays, containing one PCa and three controls. The specificity achieved was 71% and 75% for the two dogs, respectively, while the sensitivity was 13% and 25%. Those data were lower than expected by chance. Probably the drop in sensitivity and specificity registered was due to the procedure adopted for training and blind tests. In particular, during the training phase, samples from the same donors were presented to dogs several times, and this may have led dogs to memorise sample-specific odour fingerprint that they did not rediscover during the double-blind tests, since only samples from new donors were considered. Indeed, comparison of the urine sample choices made by the dogs in different tests suggested that each dog was using different odour signature for the sample selection. 

This outcome emphasises the importance of using different urine samples for the training and the double-blind tests. However, this outcome does not exclude the possibility that dogs could learn to generalise based on a common PCa with an optimised training procedure.

In order to reach this goal, Taverna et al. [19] defined a rigorous procedure for dogs’ training aimed at the identification of a pool of VOCs specific for PCa emanating from urine samples. Two German Shepherd explosion detection dogs were trained using the clicker training. The dogs were taught to sit in front of the cancerous sample after sniffing a set of six urine samples, including one PCa sample and five controls. Urine samples were stored at −20 °C. For the analysis, 2 mL of each sample were defrosted and housed in circular perforated metal containers. Metal containers were placed in thermally sealed plastic packets to avoid any contamination.

Taverna’s research involved a huge and multifaceted population (i.e., 902 participants), including also men and women suffering from different tumours. Diagnostic test performance was evaluated, considering the whole population, after excluding females, and considering only control men older than 45 years. In all cases, sensitivity was higher than 98% and specificity was over 96%.

In the attempt to resume and order the main information present in the scientific literature regarding the use of trained dogs for PCa detection in a table, according to a common logic for the readers’ use, the following categories were defined and used: involved population and trained dogs, sample preparation, training methods, and diagnostic accuracy achieved in terms of sensitivity and specificity [95,96,97,98] (Table 1).

#### 3.1.2. Electronic Nose

Given the promising results reported in studies regarding the dogs’ capability of detecting PCa by sniffing urine samples, some research groups [20,21,22,23,24] started investigating the possibility to transfer those results to an instrumental method based on the analysis of urine samples through electronic noses (EN). 

Electronic noses are already adopted in the food and pharmaceutical industries [87,99,100], in the environmental field [101] and for indoor air quality monitoring [102]. Their application to diagnostics has been studied with promising results for discrimination of bacteria cultures [103,104], or the detection of urinary tract infections [105,106,107], diabetes [108], kidney diseases [109,110], bowel diseases [111,112] by means of urine analysis, and lung and colon cancers by means of exhaled breath analysis [113].

In the field of PCa diagnosis, Bernabei et al. [20] investigated the ability of an EN based on 8 quartz crystal microbalances coated with different metalloporphyrins, i.e., the ENQBE, to characterise urine headspaces for the detection of PCa and bladder cancers (BC). The research involved 113 patients, including other urological diseases in the control group and PCa post-surgical patients. For the creation of urine headspace, the sample was kept at 25 °C for the time necessary to obtain a steady gaseous mixture. Then, 10 mL of the enriched headspace were extracted and injected in a 2 L bag pre-filled with N_2_. 

Data were processed by means of principal component analysis (PCA) and discriminant analysis solved by partial least squares (PLS-DA). The leave one out cross validation (LOOCV) was adopted to evaluate the classification performance. The PCA score plot shows a good discrimination between PCa, BC samples and controls. A very interesting result was the migration of post-surgical patients from the PCa cluster to the healthy cluster, suggesting the ability of the EN not only to detect urological diseases, but also to monitor the response to treatments.

D’Amico et al. [21] conducted a pilot study for PCa diagnosis using an EN equipped with 8 non-selective gas sensors, coated with metalloporphyrins. Urine samples were provided before prostate biopsy by 21 volunteers, including men suffering from PCa and healthy subjects. Each control participant provided two urine samples, thus executing two different trials. For the EN analysis, urine was put in a sterile urine box with a dedicated top to extract the headspace to be analysed. Data were processed by PLS-DA, and only a qualitative plot is reported for evaluating the discrimination achieved. This study needs to be enlarged in terms of population involved. In particular, the number of sick participants should be increased in order to create two olfactory classes, approximately including the same number of subjects, otherwise, the classification may be biased towards the class with most representatives. Moreover, the experimental procedure should be standardised in order to confirm results.

Asimakopoulos et al. [22] evaluated the efficacy of PCa detection by an EN equipped with 8 non-selective quartz crystal microbalance gas sensors coated with different metalloporphyrins, based on the analysis of urine samples collected before prostate biopsy. Each participant was asked to collect the initial part of the urination and the midstream in two different sterile vials. The measurements were performed without knowing biopsy outcomes. A first aspect to be highlighted is the different EN outcome related to the analyses of different parts of the urination. In particular, the analysis of midstream urine did not provide useful information for PCa detection. Conversely, the analysis of the first part of urine correlated with prostate biopsy outcomes. The author attributed this result to an increased content of elements of prostatic secretions in the first part of the urines. In this study, the EN performance achieved a sensitivity of 71.4% (CI 42–92%) and a specificity of 92.6% (CI 76–99%).

Santonico et al. [23] resumed the study of D’Amico et al. [21] and analysed urine headspaces of men suffering from PCa searching for specific odour fingerprints with the EN developed at the University of Rome “Tor Vergata”. Measurements were performed at room temperature and data were elaborated by means of PLS-DA using LOOCV. The test achieved a specificity of 93%. However, the PLS-DA score plot showed only a partial discrimination of positive samples from controls.

Roine et al. [24] evaluated the EN ability of discriminating PCa from BPH by means of the analysis of urine headspaces. They involved 50 patients with confirmed PCa, and 24 control subjects suffering from BPH, among them, 15 patients provided urine preoperatively and 9 patients 3 months postoperatively. Urines were collected in the morning, and samples were stored at −70 °C. 

The EN used was a commercial model (i.e., ChemProR 100, Environics Inc., Mikkeli, Finland), equipped with ion mobility cell consisting of 8 electrode strips and a metal oxide-based semiconductor cell. For the analysis, urine was defrosted and pipetted to a polystyrene culture plate, which was heated and maintained at 37 °C. Each measure lasted 25 min: 15 min for urine analysis and 10 min for recovery. Sensitivity and specificity were, respectively, 78% and 67%, when using LOOCV, and 82% and 88% when using LDA. Urine samples were collected only from men who required surgical operation. The authors in the conclusions suggested extending the study, considering patients with mild symptoms and the effects of other factors responsible for urine odour alterations, such as diet, medications, or hydration.

Also for this technology, we tried to resume and schematise the main information gathered from the scientific literature in a table. The logic adopted in this case involved the definition of the following categories—besides authors and year of publication—population involved, sample preparation methods, statistical methods adopted, and results achieved in terms of classification performance (Table 2).

### 3.2. Chemical Analysis

Recent years have seen remarkable progresses in the characterisation and the quantification of biological molecules and the explanation of their roles in cells, providing new pathways for diagnostic purposes [114]. Metabolic alterations may be indicative of disease incidence, and may allow for identification of cancer biomarkers [7], defined by National Cancer Institute as biological molecules found in body fluids or tissues that are signs of normal or abnormal biological process or of a condition or disease [115]. 

The discovery of new and more efficacious PCa biomarkers may improve current diagnostic procedure, allowing determination of which patients will develop an aggressive tumour, prediction of recurrence, and monitoring of response to treatments. 

In this section, we tried to provide a general overview of literature works focused on the chemical characterisation of liquid or gaseous urine aimed at early PCa detection.

Sreekumar et al. [25] proposed sarcosine, uracil, kynurenine, glycerol-3-phosphate, leucine, and proline as PCa biomarkers. They reported that sarcosine was significantly higher in urine sediments (accuracy AUC 71%) and supernatants (AUC 67%) of PCa patients, while uracil, kynurenine, glycerol-3-phosphate, leucine, and proline were elevated upon disease progression.

Jentzmik et al. [26] involved 45 healthy subjects and 107 PCa patients. They determined sarcosine levels in urine by GC-MS, using a commercial amino acid assay, and discovered that the median sarcosine/creatinine was 13% lower in PCa patients than in controls. However, ROC analyses proved the inefficacy of sarcosine as PCa biomarker in comparison with total PSA, since the discrimination between PCa patients and controls was significantly worse. Authors recognised a limit of their study in the higher proportion of PCa patients than healthy subjects.

Jiang et al. [27] developed a novel method for the quantification of six urinary metabolites suitable as PCa biomarkers, i.e., sarcosine, proline, kynurenine, uracil, glycerol-3-phosphate, and creatinine, and reported that their average concentrations were higher in PCa urine than controls.

Wu et al. [28] adopted microwave-assisted derivatisation (MAD) combined with GC-MS to analyse urine samples from 20 PCa patients, 8 patients with BHP, and 20 healthy men, and compared metabolic information. PCa patients’ average sarcosine levels were 13% higher than healthy controls and 19% higher than BPH samples. Propenoic acid, dihyroxybutanoic acid, creatinine, xylonic acid, and dihyroxybutanoic acid were proposed as PCa biomarkers, since their concentrations were higher in PCa patients with respect to controls.

Stabler et al. [29] compared markers in serum and urine of patients with rapidly recurrent prostate cancer to recurrence-free patients after radical prostatectomy. They tested methionine metabolites in urine and serum as pre-surgical markers for aggressive disease. They reported that urinary dimethylglycine and homocysteine of the groups did not differ significantly, while urinary sarcosine and cysteine were significantly higher in recurrent patients.

Bianchi et al. [30] developed and validated a SPME-GC/MS method for the analysis of urine and urinary sediments. Their results showed that sarcosine could be adopted as PCa biomarker. Correspondence of a cut-off of 179 µg_sarcosine_/g_creatinine_ sensitivity of 79% and specificity of 87% were achieved. Shamsipur et al. [31] combined dispersive derivatisation liquid–liquid microextraction (DDLLME) with GC-MS and LC-MS to define a method for the determination of PCa metabolite biomarkers, including sarcosine, alanine, leucine, and proline. They proved that sarcosine mean concentration was higher in PCa patients, while leucine mean concentration was lower.

Struck-Lewicka et al. [32] reported that metabolites significantly changed in PCa patients were mainly involved in amino acid, organic acid, sphingolipid, fatty acid, and carbohydrate metabolic pathways. Glycine, serine, threonine, alanine, indole, hippurate, hydroxyhippurate, tryptophan, kynurenate, tyrosine, indole acetate, indolelectate, quinate, phenylacetamide, glutamine, isocitrate, aconitate, succinate, sucrose, sorbose, arabinose, arabitol, inositol, galactaric acid, acetic, propanoic, propenoic, butanoic acid, dimethylheptanoyl, carnitine, propanoylcarnitine, butyrylcarnitine, octanoylcarnitine, hydroxysphinganine, and C16 sphingosine were proposed as indicators of metabolic alterations due to PCa. All these metabolites were present at lower concentrations in PCa samples with respect to controls.

Heger et al. [33] monitored the level of potential non-invasive PCa biomarkers responsible for the genesis and the progression of the tumour. PSA and free PSA were determined by immunoenzymometric assay too. They detected statistically significant differences in concentrations of amino acids (i.e., aspartic acid, threonine, methionine, isoleucine, leucine, tyrosine, arginine, sarcosine, proline) and biochemical parameters (i.e., concentrations of K^+^, uric acid, urea, and creatinine). In particular, amino acids, urea, and creatinine were more abundant in PCa patients, while K+ and uric acid concentrations were higher in controls.

Khalid et al. [34] investigated the VOCs emanating from urine samples. They proposed 2,6-dimethyl-7-octen-2-ol, pentanal, 3-octanone, 2-octanone as suitable biomarkers for PCa detection. Except for pentanal, all of these compounds were downregulated and/or less frequently present in the urine samples from PCa patients compared to healthy subjects. The accuracy of the model based on four biomarkers discovered was 63–65%, while it was 74% (RF) and 65% (LDA), if combined with PSA level.

Tsoi et al. [35] evaluated the potential role of urinary polyamines, i.e., putrescine (Put), spermidine (Spd), and spermine (Spm), in PCa development. Their levels were determined by UPLC-MS/MS. Spm demonstrated a good discrimination performance between PCa patients and BPH patients: normalised Spm showed a significant decrease in PCa patients compared to non-cancerous cases, including BPH patients. Correlations between urinary Spm had also been performed with patients’ pathologic characteristics, like age, serum PSA, creatinine content and prostate volume. However, all of them showed weak correlation with correlation coefficients <0.1.

Sroka et al. [36] proposed the LC-ESI-QqQ-MS for the quantification of amino acids and amine concentrations in urine samples from PCa patients and men with diagnosed BPH. They aimed to determine whether amino acids and amine could be used to discriminate between PCa and BPH. Arginine, homoserine, and proline were more abundant in samples from PCa patients compared to patients with benign growth. Their study underlined also that patients classified with Gleason score (GS) 7 had significantly higher concentrations of proline, homoserine, and tyramine compared with those classified as GS 6 or GS 5. They also showed the inefficacy of sarcosine as PCa indicator, by determining its levels before and after the prostate massage.

Fernandez-Peralbo et al. [37] identified 28 significant metabolites for PCa detection. Dimethyllysine, 5-acetamidovalerate, acetyllysine, trimethyllysine, imidazole lactate, histidine, methylhistidine, acetylhistidine, urea, acetylarginine, acetylcitrulline, acetylputrescine, dimethylarginine, citrulline, tyrosine, 8-methoxykynurenate, kynurenic acid, xanthurenic acid, sulfoacetate, isethionate, acetyltaurine, acetylaspartate, acetylaspartylglutamic acid, 2-oxoglutaramate, 2-pyrrolidone-5-carboxylate, 5-methyldeoxycytidine-5-phoshate, 7-methylguanosine were present at lower concentrations in PCa urine samples than controls, while 7-methylguanine reported higher concentrations in PCa patients. Those metabolites were used to develop a PLS-DA model, which achieved 88.4% and 92.9% of sensitivity and specificity, respectively.

Gkotsos et al. [38] measured sarcosine, uracil, and kynurenic acid concentrations by UPLC-MS/MS and LC-ESI-MS/MS in urine from PCa patients, men with elevated PSA serum levels, and healthy subjects. Decreased median sarcosine and kynurenic acid and increased uracil concentrations were observed in PCa samples compared to controls. The ROC curve analysis showed that sarcosine and uracil did not correlate with the clinical status of subjects considered. Contrarily, kynurenic acid seemed a suitable PCa biomarker, especially in cases where the urine was collected after prostatic massage. However, this metabolite cis not useful in order to monitor disease progression.

Derezinski et al. [39] presented a comprehensive analysis of amino acids in urine. Their study provided strong evidence those branched-chain amino acids metabolic pathways can be a valuable source of markers for prostate cancer. The univariate statistical analyses performed showed that, in PCa samples, taurine was present at significant higher level, while γ-amino-*n*-butyric acid, phosphoethanolamine, ethanolamine, homocitrulline, arginine, δ-hydroxylysine, and asparagine occurred at significantly lower levels with respect to healthy samples. Moreover, γ-amino-*n*-butyric acid, phosphoethanolamine, ethanolamine, homocitrulline, arginine, δ-hydroxylysine, asparagine, cystathionine, and methionine had AUC higher than 75%. The PLS-DA model built on urine amino acid levels achieved sensitivity and specificity of 89.47% and 73.33%, respectively, whereas the total accuracy of classification was 82.35%.

In 2015, Aggio et al. [40] came up with a particular approach based on a “hybrid” system. In particular, they proposed a GC-MOS system, comprising a GC oven fitted with a commercial capillary column interfaced with a MOS sensor working at 450 °C, for classifying urine samples from patients with urological symptoms. Their study included 58 men with PCa, 24 with BC, and 73 with haematuria and/or poor stream, without cancer. The headspace was injected into the inlet of the GC-sensor system, and the gas sensor signals were processed by PCA to visualise the discrimination achieved. LDA and support vector machine (SVM) were used as statistical models to diagnose unknown samples. The performance of the classifiers was validated by LOOCV, repeated 10FoldCV, repeated DoubleCV, and Monte Carlo permutations. The first two principal components of the PCA performed on dataset relevant to PCa and control samples show a good discrimination between the two classes. In particular, the sensitivity reached was higher than 93%, while the specificity was above 95%.

The same work of schematisation of the significant data and information contained in the—in this case quite rich numbers of—scientific literature, was done for the papers regarding the application of chemical analyses for PCa detection. Table 3, besides authors and years, reports the population involved, the sample preparation methods, the analytical methods, the statistical methods, and the biomarkers proposed.

## 4. Discussion and Conclusions

In the previous paragraphs, we tried to give an overview of the innovative techniques that are being studied in the field of PCa diagnosis. This section has the aim of critically discussing the different methods proposed, and to compare them in terms of advantages, drawbacks, and future perspectives.

In general, innovative tools proposed in recent years can be grouped into two macro-categories, according to the method adopted. Many authors explored the possibility of analysing urine odour fingerprint, while others preferred the chemical characterisation of liquid urine or of its gaseous headspace, trying to identify potential PCa biomarkers. All approaches propose the comparative analysis of urine samples from healthy men and PCa patients with the aim of discriminating the two classes.

Studies reporting the adoption of trained dogs for PCa detection proved its feasibility and the diagnostic accuracy achieved in terms of sensibility and specificity was promising. However, they boosted further investigation, since some critical issues about the definition of the experimental protocol emerged. In particular, the type of training and blind test, the training site, the team of trainers involved, the frequency and duration of training, the method adopted for the sample somministration and the number of blind-test runs influence dogs’ discriminative ability. In addition, it is worth considering the high costs related to dogs’ training and the effect of the training procedure on the classification performance. 

Considering the limits related to the adoption of trained dogs for the development of a large-scale diagnostic tool, researchers started investigating the possibility to transfer those experimental observations to an instrumental method based on the analysis of urine samples through electronic noses. 

All literary works, summarised in Table 2, reporting the adoption of the EN for PCa diagnosis, focused on the characterisation of urine headspace. In general, published results confirm the capability of the EN to distinguish between urine samples collected from men suffering from PCa and healthy subjects, with very promising diagnostic accuracy in terms of specificity and sensitivity, although there is no uniformity concerning sample preparation, analysis, and data processing techniques among the different studies proposed. 

It is worth considering that none of the abovementioned studies address the problem of sensor response drift over time, and repeatability among different instruments. Sensor drift (i.e., non-deterministic temporal variations of the sensor response when exposed to the same analytes under identical conditions) is recognised as one of the main problems associated with gas sensor [116]. This aspect limits the EN ability to operate over long time periods in all fields of application of ENs, thus, it is one of the key criticalities to be solved for the development and spread of an innovative PCa diagnostic tool based on EN analysis.

Recent advances in the understanding of cancer genesis and progression and in the characterisation and the quantification of biological molecules boosted the research in the field of urine chemical characterisation for PCa diagnostic purposes. Indeed, many research groups started working on the identification of novel biomarkers able to improve the diagnostic and prognostic accuracy of the traditional tests.

Most of investigated literary works, summarised in Table 3, proposed the comparative analysis of samples from PCa patients and controls, since authors agreed that PCa development caused alterations of different metabolic pathways, such as amino acid, fatty acid, and carbohydrate metabolism. They developed diverse methods, combining different analytical techniques, for the detection and quantification of changes in metabolites levels in PCa samples compared to healthy ones. Sometimes, different stages of the tumour were considered to evaluate also the efficacy of staging of proposed PCa biomarkers. 

Nevertheless, the in-depth analysis of these literary works highlighted that no exhaustive results have been published until now, since many different metabolites were proposed as suitable PCa biomarkers, and divergent opinions upon the same metabolites emerged in different studies. 

Many literary works considered proposed a quantitative characterisation of urine samples. Table 4 reports concentrations trends of proposed biomarkers in cancerous samples compared to controls, as reported in literature.

Among the proposed biomarkers, the most debated is sarcosine. Indeed, many authors [25,28,29,30,31] reported that its level in urine from PCa patients is higher than in control samples, and that its classification performance is good, whereas other researchers [36,38] showed that changes in its concentrations between PCa and healthy men were not statistically significant.

Isoleucine, threonine, methionine, tyrosine, arginine, and kynurenic acid trends in PCa and control groups were debated too. In fact, Struck-Lewicka et al. [32], Fernandez-Peralbo et al. [37], and Derezinski et al. [39] reported lower levels in PCa samples compared to controls, while Heger et al. [33], Jiang et al. [27], and Sroka et al. [36] reported opposite trends. 

Proline, citrulline, and homocitrulline seemed to be the most suitable biomarkers for PCa detection, since many authors [27,31,33,36,37,38] agreed that their levels were higher in PCa samples compared to control ones, and classification performance of models built on these metabolites was encouraging.

Future works should focus on those disparities among different studies, in order to adopt chemical analyses with the objective of improving traditional diagnostic procedures.

The key aspects of different approaches discussed in this section were schematically compared in terms of pros and cons as reported in Table 5.

Considering the specific and different difficulties associated with each of the discussed innovative diagnostic approaches, possibly the evolution towards “hybrid” systems combining two or more different approaches, as proposed by Aggio et al. [40], might represent an answer to those contradictory outcomes in the next future. 

It is realistic to think of a possible future development of a “hybrid” system based on the combination of the odour analysis performed by the EN with the chemical characterisation of urine samples, which should differ from previous studies in this field by focusing on odour analysis, and take unique advantage of the olfactory differences highlighted by EN analysis. This might possibly focus on the identification of those compounds responsible for the alteration of urine odour, thus simplifying the task of the chemical characterisation of urine and provide an innovative pathway for the discovery of new and more efficient biomarkers specific for the PCa.

In general, one aspect that is common to all innovative diagnostic methods is the importance of the size of the population involved. In particular, the classes considered (i.e., PCa patients and healthy subjects) should include approximately the same number of samples, otherwise, the output of the innovative tests tend to be biased towards the class with most representatives [117]. Almost all literary works here presented involved small populations and, in many cases, the control and the PCa groups were not numerically comparable. 

Another very important critical point is that the majority of studies do not discuss the specificity of the method towards PCa with respect to other pathologies. Indeed, it would be very interesting to investigate the specificity to other types of tumours, and especially, tumours associated with the urinary tract (i.e., bladder and kidney cancers). This aspect is particularly important for the development of a specific diagnostic tool whose answer should be ideally positive only in cases of PCa, and negative for any other disease, as obtained by Taverna et al. [19] in his experience with trained dogs. 

Only Bernabei et al. [20] and Aggio et al. [40] tested the their methods towards another urological tumour, i.e., the bladder cancer. Bernabei et al. [20] reported a PLS-DA score plot that showed the complete discrimination between healthy and sick subjects and a PCA score plot, where the gradual discrimination between different cancers is visible. On the contrary, Aggio et al. [40] reported only the performance of the EN in distinguishing between controls and BC or PCa separately.

Roine et al. [24], Wu et al. [28], Bianchi et al. [30], Tsoi et al. [35] and Sroka et al. [36] included in their chemical comparative studies men suffering from BPH, which typically causes a high number of false positives in traditional procedures. However, their results should be confirmed by considering larger populations.

One aspect common to the different methods proposed, which is worth highlighting here as part of the critical discussion, is the importance of the training phase, which should be intended as the effective training of dogs for developing the discriminative ability between different urine samples, and for the development of a suitable model for data processing and classification for senso-instrumental and chemical approaches.

According to the scientific literature here examined, it is not possible to identify a data processing or classification algorithm whose classification performance clearly prevails compared to others. Indeed, the proposed algorithms are sometimes very different from each other (e.g., PCA, PLS-DA, ROC analysis, Mann–Whitney analysis, Pearson correlation) and this result in the variability of classification performances reported. However, it is worth highlighting that mathematics, even when involving extremely elaborate and complex algorithms, can never adjust bad data [118]. Therefore, it is very important to optimise input data, especially when samples are characterised by high variability, as with urine. This is a crucial aspect of this research field aimed at the development of a large-scale PCa diagnostic tool. 

Last but not least, a fundamental aspect for the development of a method that might become of widespread use in clinical diagnosis is the switch from a complex laboratory apparatus to an easy-to-use instrument. The training of dogs, the use of electronic noses or of chemical analysers, require highly specialised personnel, whereas the field of clinical diagnosis is rapidly evolving towards point-of-care tests, which ideally should be used by non-specific staff. This is a great challenge for current research in the field of medical diagnostics.

Despite the difficulties associated with the development of innovative and reliable diagnostic techniques, a significant increase of the research in this field—and hopefully the successful introduction of some of these techniques in clinical diagnosis—in the near future is to be expected, due to the high social and economic impact that new technologies for early diagnosis of cancer might have in today’s culture.

As a last consideration, it might be worth highlighting that, given the ambitious purpose, only a multidisciplinary team, that includes clinicians, engineering, biologists, physicians, and biochemical scientists, collaborate together to understand the complexity of human beings. This way of thinking may further help to clarify concepts and indicate alternative experiments in order to develop appropriate diagnostic methods [42].

## Figures and Tables

**Table 1:** Literary studies proposing trained dogs for urine analysis for early prostate cancer (PCa) detection.

Reference	Authors	Population Involved Controls/Sick		Trained Dogs	Samples Collection and Treatments	Training Method	Results
[16]	Gordon et al. (2008)	186	57 PCa	4	Storage temperature: −20 °C; Sample preparation: thawed, placed in a screw-top vial; Sample somministration: screw-top vials were put into mason jars	Training site: trainer’s home Trainer: Owner Duration: 12–14 months Frequency: 2–7 d/w	Specificity: 1: 36%; 2: 36%; 3: 63%; 4: 81% Sensitivity: 1: <10%; 2: <20%; 3: 20%; 4: 25%
[17]	Cornu et al. (2010)	training phase: 16; double blind phase: 33	training phase: 26; double blind phase: 33	1	Storage temperature: −4 °C; Sample preparation: slowly heating to 37 °C; Sample somministration: samples placed in perforated boxes	Trainer: Professional Duration: 16 months Frequency: 5 d/w	Specificity: 91%; Sensitivity: 91%
[18]	Elliker et al. (2014)	67	50 PCa	2	Storage temperature: −20 °C; Sample preparation: defrosting in a 37 °C water bath; Sample somministration: samples were put in open top propylene test tubes	Stage 1: dogs had to find and indicate PCa urine samples; Stage 2: dogs had to discriminate PCa samples from controls; No information about duration and frequency of training	Specificity: 1: 71%; 2: 75%; Sensitivity: 1: 13%; 2: 25%
[19]	Taverna et al. (2015)	540	362 PCa	2	Storage temperature: −20 °C; Sample preparation: defrosting to 37 °C; Sample somministration: samples were put into circular perforated metal containers placed in thermally sealed plastic containers	Training Site: central Trainer: professional No information about duration and frequency of training	Specificity: 1: 98.7%; 2: 97.6%; Sensitivity: 1: 100%; 2: 98.6%

**Table 2:** Literary studies proposing electronic nose (EN) adoption for PCa diagnosis through urine analysis.

Reference	Authors	Participants Controls/Sick		Samples Collection and Treatments	Instrument (Sensor Type)	Statistical Methods	Results
[20]	Bernabei et al. (2007)	29 BPH; 33 other urological pathologies; 18 controls	25 BC 12 PCa	Urine collection: in the morning before any food intake; Storage temperature: no info; Headspace creation: urine was put at 25 °C for the necessary time to obtain a steady headspace, then 10 mL of headspace were injected into a 2 L sterile bag pre-filled with N_2_; EN analysis: no info	ENQBE (Conducting polymers)	PLS-DA; PCA; LOOCV	qualitative plot; discrimination between PCa and BC samples and controls 100%; differentiation between different classes, not complete discrimination
[21]	D’Amico et al. (2012)	15	6 PCa	Urine collection: before PBs; Storage temperature: no info; Headspace creation: a dynamic headspace was obtained putting urine in sterile urine boxes with a dedicated top; EN analysis: no info	EN: University of Rome “Tor Vergata” (Conducting polymers)	PLS-DA	qualitative plot
[22]	Asimakopoulos et al. (2014)	27	14 PCa	Urine collection: before PBs; Storage temperature: no info; Headspace creation: a dynamic headspace was obtained putting urine in sterile urine boxes with a dedicated top; EN analysis: no info	EN: University of Rome “Tor Vergata” (Conducting polymers)	PLS-DA; LOOCV	Sensitivity: 71.4%, specificity: 92.6%
[23]	Santonico et al. (2014)	27	14 PCa	Urine collection: before PBs; Storage temperature: no info; Headspace creation: a dynamic headspace was obtained putting urine in sterile urine boxes with a dedicated top; EN analysis: 200 s for the measurement phase, 600 s for the cleaning phase	EN: University of Rome “Tor Vergata” (Conducting polymers)	PLS-DA; LOOCV	qualitative plot
[24]	Roine et al. (2014)	24 (15 BPH and 9 post radical prostatectomy)	50 PCa	Urine collection: in the morning; Storage temperature: −70 °C; Headspace creation: urine was defrosted and pipetted to a plate heated and maintained at 37 °C; EN analysis: 15 min for the measurement phase, 10 min for recovery	EN: ChemPRO 100-eNose (Electrode strips and MOS sensors)	LOOCV; LDA	LOOCV: sensitivity 78%, specificity 67%, accuracy 77% LDA: sensitivity 82%, specificity 88%

**Table 3:** Literary studies proposing different analytical techniques for PCa biomarker identification.

Reference	Authors	Population Controls/Sick	Sample Preparation Method	Analytical Method	Statistical Methods	Biomarkers	Results
[25]	Sreekumar et al. (2009)	51/59	Urine collection: After DRE for PCa patients; Storage and pre-treatments: Samples were stored at −80 °C until analysis; Sample preparation: Samples underwent organic and aqueous extractions. The extracted was equally divided into LC and GC fractions, which were dried on a TurboVapR. Prior to injection, all samples were resuspended in identical volume and injection standards were added.	LC-MS: The vacuum dried sample was re-solubilised in 100 µL of injection solvent. The system was operated using a gradient of acetonitrile. The columns were maintained in temperature-controlled chambers during use and were exchanged and washed after every 50 injections. GC-MS: The column used was 5% phenyl-methyl polysiloxane, the temperature from 40 °C to 300 °C in 16 min. ID GC-MS: For analysing sarcosine and alanine, residual water was removed by forming an azeotrope with 100 uL of DMF and drying the suspension under vacuum. An Agilent 6890N GC equipped with a 15 m DB-5capillary column interfaced with an Agilent 5975 MSD mass detector.	Wilcoxon rank-sum test; *t*-test; Kruskal–Wallis test; Pearson’s correlation; NOVA; Z-score plot; heat maps	Sarcosine; Uracil; Kynurenine; Glycerol-3-phosphate; Leucine; Proline	Sarcosine was significantly higher in urine sediments (AUC 71%) and supernatants (AUC 67%) of PCa patients; Uracil, Kynurenine, Glycerol-3-phosphate, Leucine, Proline were elevated upon disease progression.
[26]	Jentzmik et al. (2010)	45/107	Urine collection: after DRE for PCa patients; Second morning void urine for healthy participants; Storage and pre-treatments: Samples were centrifuged (1500× *g*, 10 min, 4 °C) and stored at −80 °C; Sample preparation: no info	Ez:fast amino acid analysis: SPME followed on a L-LE with the subsequent GC-MS on a 5973 MS and 6890 GC system. Recovery was checked with samples spiked with known amounts of sarcosine.	Mann–Whitney U test; Wilcoxon matched-pairs test; Spearman rank correlation; Fischer’s exact test; ROC analysis	Sarcosine	Median Sarcosine/creatinine was 13% lower in PCa patients than in controls
[27]	Jiang et al. (2010)	5/5	Urine collection: no info; Storage and pre-treatments: Samples were frozen at −80 °C; Sample preparation: Samples were thawed at room T and diluted 3 times using water; 10 µL of diluted urine were mixed with 10 µL of the internal standard solution and 1480 µL of 0.1% formic acid in water; those samples were diluted 450 times and injected for HPLC/MS/MS analysis	HPLC: An LC system working at 25 °C under a flow rate of 250 μL/min using a gradient system with the mobile phase consisting of (A) 0.1% formic acid in water and (B) 0.1% formic acid in acetonitrile (100%) was used for metabolite separation. The gradient program was initial 98% A and 2% B, linear gradient to 60% A and 40% B in 5 min, and return to initial conditions in 0.1 min at a flow rate of 250 μL/min, followed by equilibration for 10 min. MS/MS: An API 4000Q trap MS/MS system operated in multiple-reaction monitoring mode with ESI-positive ionisation was used. Turbo Spray was used as the ion source. The capillary voltage was set at 5.5 kV. Nitrogen gas was used as the curtain gas and cone gas. The cone gas flow was 50 L/h, and the desolvaation gas flow was 800 L/h. Optimal detection conditions were determined by direct infusion of each standard solution (20 ppb) in solvent A using a syringe pump. Parent-ion and daughter-ion scans were performed using nitrogen as the collision gas at a pressure of 3.8 × 10^3^ millibar and a flow of 0.2 mL/min.	Multivariate statistics	Sarcosine; Proline; Kynurenine; Uracil; Glycerol-3-phosphate; Creatinine	nM_metabolites_/µM_creatinine_: PCa patients: Sarcosine 120; Proline 40; Kynurenine 15; Uracil 10; Glycerol-3-phosphate 85; Controls: Sarcosine 30; Proline 5; Kynurenine 8; Uracil 5; Glycerol-3-phosphate 30
[28]	Wu et al. (2010)	8 BHP; 20 healthy male/20	Urine collection: first morning urine; Storage and pre-treatments: Samples were centrifuged within 1 h at 3000 rpm for 10 min at 25 °C; aliquoted in 1 mL and stored at −80 °C; Sample preparation: Samples were thawed by incubation at 37 °C for 3 min and vortex-mixed for 15 s. 800 µL methanol, 100 µL ribitol and 100 µ were added into each sample and vortex-mixed for 5 min and ultrasonicated at room T for 5 min. pH was adjusted to 9–10 with NaOH and solution was filtered by 0.45 µm membrane. 100 µL of filtrate were transferred to a screw vial and evaporated under N_2_	ID GC-MS: 1 µL of derivatised sample was injected splittless into an Agilent 6980 GC equipped with a 30 m × 0.25 nm i.d. fused-silica capillary column with 0.25 µm HP-5MS stationary phase. Injector T was set at 250 °C, column T was initially kept at 80 °C for 3 min and increased to 280 °C at 10 °C/min, where it was held for 2 min. Column effluent was introduced into Agilent 5973 mass selective detector: quadrupole T 150 °C, ion source T 230 °C, solvent delay 180 s.	Two-sample *t* test; PCA; ROC analysis	Sarcosine; Propenoic acid; Pyrimidine; Dihyroxybutanoic acid; Creatinine; Purine; Glucopyranoside; Ribofuranoside; Xylonic acid; Xylopyranose	PCa patients average sarcosine value were 13% higher than healthy controls and 19% higher than BPH controls. Also propenoic acid, dihyroxybutanoic acid, creatinine, and xylonic acid, dihyroxybutanoic acid and xylonic acid, concentrations were higher in PCa patients.
[29]	Stabler et al. (2011)	29 recurrent free; 25 PCa recurrence	Urine collection: before prostatectomy; Storage and pre-treatments: Samples were stored at −80 °C; Sample preparation: no info	GC-MS: A Durabond DB.1 fused silica capillary column (30 m × 0.25 mm) from J&W Scientific, Inc. and a Hewlett-Packard Co. 5992B gas chromatograph-mass spectrometer equipped with a falling needle injector were used.	Wilcoxon rank sum test; Fisher exact test; Spearman’s rank correlation	Cysteine; Homocysteine; Dimethylglycine; Sarcosine	Higher serum homocysteine, cystathionine, and cysteine levels independently predicted risk of early biochemical recurrence and PCa aggressiveness. The methionine further supplemented known clinical variables to increase sensitivity and specificity.
[30]	Bianchi et al. (2011)	13 healthy; 10 BHP/33	Urine collection: after DRE; Storage and pre-treatments: no info; Sample preparation: no info	SPME: A Gerstel MultiPurpose Sampler DualRail WorkStation MPS autosampler equipped with two sample trays, two-heated incubator shakers, a 100 µL syringe and a 3-position trays MFX was used. Hexyl chloroformate (10 μL), 10 μL of pyridine and 10 μL of hexanol were added, under continuous agitation at 500 rpm, in 0.9 mL clear crimp vials with sleeve for 10 × 32 vial containing 400 μL of urine. Norvaline was used as internal standard. After 5 min, 20 μL were diluted in 0.9 mL clear crimp vials previously filled with 800 μL of water. Simultaneously, the SPME fibres were transported between the 3-position tray and the vial. Urine sediments were washed with water under sonication and filtered. The filter was then broken up adding 500 µL of HCl and 1 mL of acetone into a 10 mL vial placed in an ultrasound bath for 10 min. Extraction was performed using PDMS/DBV fibre that was immersed in vial for 15 min at 35 °C. A constant magnetic stirring was applied. The desorption was carried out at 260 °C for 1 min. GC-MS: Oven setting was as follows: 80 °C for 0.3 min, 80 °C min^−1^ up to 200 °C, 200 °C for 0.3 min, 50 °C min^−1^ up to 290 °C. Inlet pressure, column flow and average linear velocity were 623.1 kPa, 0.97 mL min^−1^ and 51.3 cm s^−1^. The QP 2010 series MS detector (Shimadzu) equipped with the acquisition system GC Solution software was operated under the selected ion monitoring mode by applying a delay time of 2.9 min	Mann–Whitney U; Kruskal–Wallis tests; ROC analysis	Sarcosine; N-ethylglycine	µg_Sarcosine_/g_Creatinine_ discriminates between healthy, BHP and PCa patients Cut-off 179 µg/g: sensitivity 79%; specificity 87%
[31]	Shamsipur et al. (2012)	20/12	Urine collection: no info; Storage and pre-treatments: sample were frozen at −22 °C; Sample preparation: urine was thawed at room T and shaken vigorously for 1 min	DDLLME: 4 mL of water spiked with standard solution were treated with 12 M NaOH to obtain the desired pH. Standard amino acids were spiked into the solution at a level of 200 µg/L for initial screening and 50 µg/L for final optimisation. 150 µL acetonitrile, 200 µL pyridine and 25 µL carbon tetrachloride were added and the solution was mixed vigorously for 15 s. i-BuCF (250 µL) were added and shaken for 30 s. The solution was left to stand for 1 min and then centrifuged at 2260× *g* for 4 min for phase separation. 10 µL of the sediment phase was injected into the GC-MS for analysis. GC-MS: Processed samples were analysed using an Agilent 6890 GC coupled to an Agilent 5973 inert EI/CI mass selective detector. He was maintained at a constant flow of 1.8 mL min^−1^. The injection port was set to splitless and maintained at an optimised temperature of 280 °C. The oven temperature program was as follows: 80 °C (initial temperature), ramped to 155 °C at 10 °C min^−1^, holding at 155 °C for 5 min, then ramped to 172 °C at 2 °C min^−1^ holding for 2 min, finally ramped to 280 °C at 40 °C min^−1^ and holding for 6 min. T settings for the transfer-line heater, ion source, and quadrupole of the MS were 280, 150, and 150 °C, respectively. The dwell time for each scan was 150 ms ion^−1^, and the solvent delay was 7 min. The electron impact ionisation energy was 70 eV.	Bland-Altman	Sarcosine; Alanine; Proline; Leucine	Sarcosine mean concentrations were higher in PCa patients; Leucine mean concentration was lower in PCa patients
[32]	Struck-Lewicka et al. (2014)	32/32	Urine collection: no info; Storage and pre-treatments: Samples were stored at −80 °C; Sample preparation: no info	LC-TOF/MS: Urine samples after thawing at room temperature were vortex-mixed for 1 min and centrifuged at 4000× *g* for 10 min. Subsequently the supernatant was diluted in deionised water and then centrifuged at 4000× *g* for 15 min. After centrifugation, the samples were filtered directly to HPLC vials using 0.2 μm nylon filters. GC-MS: samples were thawed at room temperature for 1 h. The first step was addition of 50 μL of urease to 200 μL of urine. Next, the sample incubation in 37 °C for 30 min was applied (to decompose and remove excess amount of urea). Next, 800 μL of cold methanol (kept for 30 min in −80 °C) and 10 μL of pentadecanoic acid were added to urine samples. Then the samples were vortex-mixed for 5 min and centrifuged at 4000 *g* for 15 min. 200 μL of supernatants were transferred into glass inserts in GC vials and evaporated to dryness in 30 °C for 1 h 30 min. Next, 30 μL of methoxyamine in pyridine in concentration of 15 mg/mL was added to urine samples. The next step was vortex-mixing of each sample for 10 min and then incubation of all samples for 16 h in room temperature in dark place. The silylation process was performed with addition of 30 μL of BSTFA with 1% TMCS, vortex-mixing of each sample for 5 min and incubation for 1 h in 70 °C. Before GC-MS analysis, addition of 70 μL of hexane and vortex-mixing for 10 min were performed	MFE algorithm; PCA; PLS-DA; 7-fold cross validation	35 metabolites	LC-TOF/MS: Positive ionisation mode R2 0.756, G2 0.579; Negative ionisation mode R2 0.763, G2 0.508 GC-MS: R2 0.788, G2 0.711
[33]	Heger et al. (2014)	32/32	Urine collection: no info; Storage and pre-treatments: 500 µL of urine were mixed with 500 µL of 35% HCl and mineralised using the microwave equipment MW 3000. 100 µL of mineralised sample were diluted with 900 µL of dilution buffer and centrifuged using Centrifuge 5417R. 500 µL of the sample were diluted in 500 µL of 0.6 M NaOH; Sample preparation: no info	IELC: A glass column with an inner diameter of 3.7 and length of 350 mm was filled manually with strong cation exchanger in sodium cycle with ~12 μm particles and 8% porosity. The column was thermostated at 60 °C. Double channel VIS detector with an inner cell of 5 μL was set to two wavelengths: 440 and 570 nm. Elution of amino acids was carried out by buffer containing 10.0 g of citric acid, 5.6 g of sodium citrate, and 8.36 g of natrium chloride per litre of solution (pH 3.0). The flow rate was 0.25 mL·min^−1^. The reactor temperature was set to 120 °C. IEMA: The immunoenzymometric assay was used for analysis of PSA and fPSA	Shapiro–Wilk test; *t* test; hierarchical clustering	aspartic acid, threonine, methionine, isoleucine, leucine, tyrosine, arginine; sarcosine; proline; concentrations of K+, Na+, Cl−, uric acid, urea, PSA, glucose, total proteins, fPSA, creatinine and pH	All amino acids were increased in PCa patients, except for phenylalanine amounts. In controls, higher levels of K+ and uric acid and lower levels of urea and creatine were detected. PSA and free PSA were below the detection limit in controls.
[34]	Khalid et al. (2015)	43/59	Urine collection: no info; Storage and pre-treatments: Samples were stored at −20 °C; Sample preparation: Each sample was defrosted by immersing the vial in a water bath at 60 °C for 30 s. One single aliquot of urine sample per patient was used for VOC analysis. Thereafter, each sample was treated with an equal volume (0.75 mL) of sodium hydroxide 1 M. The mixture was equilibrated at 60 °C in a water bath for 30 min prior to SPME.	SPME: The SPME fibre was 85 μm thick and consisted of carboxen/polydimethylsiloxane. It was exposed to the headspace above the urine mixture for 20 min. GC-MS: VOCs were thermally desorbed from the fibre at 220 °C in the injection port of the GC/MS for 5 min. Injection was made in splitless mode and a split of 50 mL/min was turned on two minutes into the run. It was used helium as carrier gas (99.996% purity). Capillary column consisted of 94% dimethyl polysiloxane and 6% cyanopropyl-phenyl. The GC/MS T program of the run was as follows: initial oven T was held at 40 °C for 2 min then T was ramped up at a rate of 5 °C/min to 220 °C, with a 4 min hold at this T to give a total run time of 42 min. The mass spectrometer was run in electron impact (EI) ionisation mode, scanning the mass ion range 10–300 at 0.05 scan/s. A 4 min solvent delay was used at the start of the run.	Random Forest; LDA; 10-fold cross validation; double cross validation	2,6-dimethyl-7-octen-2-ol; Pentanal; 3-octanone; 2-octanone	Except for pentanal, all of these compounds were down-regulated and/or less frequently present in the urine samples from PCa patients. Model AUC based on 4 biomarkers discovered was 63–65%, while it was 74% (RF) and 65% (LDA) if combined with PSA level.
[35]	Tsoi et al. (2016)	88 BHP; 11 healthy/66	Urine collection: after lunch prior PBs; Storage and pre-treatments: −20 °C; Sample preparation: Firstly, urine samples were thawed and centrifuged for 5 min at 13,000 rpm at room T. Urine sample supernatant (120 μL) and 60 μL of internal standard working solution were mixed with 420 μL of water. Of this well-mixed solution, 550 μL was passed through SPE, which had been conditioned and equilibrated with 1 mL of methanol and water respectively. Water (450 μL) was passed through the cartridge afterwards to elute out all polyamines. Of these SPE-treated samples, 400 μL were then mixed with 100 μL of 10% HFBA, and the final mixture was ready for instrumental analysis	UPLC-MS/MS: The column used was an Agilent EclipsePlus C18 RRHD (2.1 × 50 mm, 1.8 μm) protected with an Agilent SB-C18 guard column (2.1 × 5 mm, 1.8 μm). The LC elution profiles were optimised as follows: Eluent A was water with 0.1% HFBA while eluent B was acetonitrile with 0.1% HFBA. Eluent A was decreased from 95% to 60% in 10 min, and from 60% to 10% in 1 min. Afterwards the gradient was held constant for 5 min. The gradient was then increased from 10% to 95% in 1 min, and held constant for 8 additional minutes. The autosampler and column temperatures were set at 4 and 35 °C respectively. Injection was achieved by 5-s needle wash in Flush Port mode for 3 times with eluent B. Ten microlitres was injected each time. For the source parameter, drying gas (N_2_) temperature was set as 300 °C with 5 L/min flow rate. Nebuliser pressure was 45 psi. Sheath gas temperature was set as 250 °C with 11 L/min flow rate. Capillary voltage was set as 3500 V.	Student’s *t*-test; ROC analysis	putrescine (Put), spermidine (Spd) and spermine (Spm)	Normalised Spd was significantly lower in PCa than in BHP patients and controls The AUC for normalised Put, Spd and Spm were found to be 0.63 ± 0.05, 0.65 ± 0.05 and 0.83 ± 0.03 respectively
[36]	Sroka et al. (2016)	25 BHP/25	Urine collection: prior and after prostate massage; Storage and pre-treatments: Sodium azide solution was added. Samples were stored at −80 °C; Sample preparation: 10 µL aliquot of each urine sample or standard solution was added to 70 µL of 200 mM borate buffer containing 25 µM 2-Aminobutyric acid, 1 mM ascorbic acid and 10 mM TCEP. The solution was vortexed, centrifuged. 20 µL of 10 mM Aqc reagent dissolved in 100%ACN was added. The solution was vortexed, centrifuged, heated with shaking at 55 °C for 10 min.	LC-ESI-QqQ-MS: Mobile phase consisted of (A) 0.1% formic acid in water (v/v) and (B) 0.1% formic acid in ACN (v/v). Flow rate was set to 300 µL min^−1^. Separation was performed at 30 °C with monitored pressure below 400 bar. Analysis time was 19 min. The gradient was run from 0–2 min using 1% solvent B, then linearly raised over 7 min from 1% to 15% solvent B. then raised to 30% solvent B over 5 min and dropped to 1% for re-equilibration which lasted 5 min. Concentrations were quantified using Agilent 1200 LC-system coupled to an Agilent 6410 ESI-QqQ-MS. Injection volumes of 2 µL of samples or standards were used. Ions were monitored in the positive ion mode. Source conditions were set to sheath gas temperature 315 °C. Gas flow 10 L min^−1^. nebuliser pressure 45 psi and capillary voltage 3800 V.	*t*-test; U Mann-Whitney analysis; ROC curves	Arginine; Homoserine; Proline; Tyramine	In PCa samples, higher concentrations of arginine both before (*p* = 0.018) and after (*p* = 0.009) prostate massage and higher levels of proline only after prostate massage (*p* = 0.032) were detected. Higher levels of proline and homoserine and tyramine correlate with GS7 with respect to GS 6 and GS 5.
[37]	Fernandez-Peralbo et al. (2016)	42/62	Urine collection: prior PBs Storage and pre-treatments: Samples were stored at −80 °C Sample preparation: After thawing at room T, urine samples were vortex-mixed for 1 min and centrifuged at 21,000× *g* for 5 min. Then, 50 μL of the supernatant were 1:2 (v/v) diluted with 5 mM ammonium formate in water (pH 5.5–7.5)	LC-QTOF: A Mediterranea Sea C18 analytical column thermostated at 25 °C was used. The initial mobile phase was a mixture of 98% phase A (0.1% formic acid in water) and 2% phase B (0.1% formic acid in ACN). After injection, the initial mobile phase was kept under isocratic conditions for 1 min; then, a linear gradient of phase B from 2% to 100% was applied within 16 min. The flow rate was 0.6 mL/min. The total analysis time was 17 min, and 5 min were required to re-establish the initial conditions. The injected volume was 5 μL. The autosampler was kept at 4 °C to increase sample stability.	unpaired *t*-test (*p*-value < 0.05); PLS-DA	28 metabolites	Almost all metabolites were present at lower concentrations in PCa patients than in controls, Training: Specificity 92.9%; Sensibility 88.4% Validation: Specificity 78.6%; Sensibility 63.2%
[38]	Gkotsos et al. (2017)	49/52	Urine collection: second morning void midstream; Storage and pre-treatments: −80 °C after post-centrifugation (each sample centrifuged at 1500× *g*, for 10 min, at 4 °C); Sample preparation: 100 μL of sample was diluted with 100 μL of MeOH. The samples were vortex-mixed (1 min) and centrifuged for 10 min (7000 *g*) to remove particulate matter and macromolecules. 50 μL of supernatant was diluted with 100 μL of MeCN and transferred to LC/MS vial, which was maintained at 10 °C.	UPLC-MS/MS: Separation was performed on a ACQUITY UPLC™ BEH AMIDE column 1.7 μm, 2.1 mm × 150 mm suitable for polar metabolites. Sarcosine, uracil, and kynurenic acid were detected using Multiple Reaction Monitoring (MRM) mode in a single injection of 15.5 min. The MRM transitions for the three metabolites were set as follows: sarcosine *m/z* 90–44, CV = 20 V, CE = 8 V; uracil *m/z* 113–70, CV = 40 V, CE = 15 V; and kynurenic acid *m/z* 190–172, CV = 32 V, CE = 12 V. For chromatographic separation the mobile phase was a mixture of (A) ACN/H_2_O, 95:5 v/v and (B) H_2_O/ACN, 70:30 v/v both with final ammonium formate buffer concentration of 10 Mm and elution was performed with a gradient program started with 100% A, then rising to 15% B linearly over the next 2 min, finally reaching 40% B over 2 min and returning to initial conditions over 5 min. The column was equilibrated for 6 min in the initial conditions. Flow rate was 0.5 mL/min	Kruskal–Wallis test; ROC analysis; Pearson correlation; Orthogonal Projections to Latent Structures Discriminant Analysis (OPLS-DA)	Sarcosine; Uracil; Kynurenic acid	Decreased median sarcosine and kynurenic acid and increased uracil concentrations were observed for patients with prostate cancer compared to participants without malignancy.
[39]	Derezinski et al. (2017)	40/49	Urine collection: second morning void midstream; Storage and pre-treatments: −80 °C after post-centrifugation (each sample centrifuged at 1500× *g*, for 10 min, at 4 °C); Sample preparation: 100 μL of sample was diluted with 100 μL of MeOH. The samples were vortex-mixed (1 min) and centrifuged for 10 min (7000 *g*) to remove particulate matter and macromolecules. 50 μL of supernatant was diluted with 100 μL of MeCN and transferred to LC/MS vial, which was maintained at 10 °C.	LC-ESI-MS/MS combined with aTRAQ: HPLC instrument 1260 Infinity combined with a 4000 QTRAP mass spectrometer with an EI source. The column was maintained at 50 °C with a flow rate of 800 μL/min. A mobile phase gradient of eluent A (0.1% formic acid and 0.01% heptafluorobutyric acid in water) and eluent B (0.1% formic acid and 0.01% heptafluorobutyric acid in methanol) was applied. The gradient profile was the following: from 2% to 40% of B from 0 till 6 min, maintained at 40% of B for 4 min, then increased to 90% of B till 11 min and held at 90% of B for 1 min. After 12 min the gradient decreased to 2% of B. From 13 to 18 min the mobile phase composition was unaltered. The injection volume was set at 2 µL. The mass spectrometer operated in positive ionisation mode with the following parameters: entrance potential, 10 V; declustering potential, 30 V and collision cell exit potential, 5 V. Collision energy of 30 eV was applied with the exception of cystathionine, cysteine, homocysteine, argininosuccinic acid, hydroxylysine, lysine, and ornithine (50 V). Scheduled multiple reaction monitoring mode was used with nitrogen as a collision gas. Data acquisition and processing were performed using the Analyst 1.5 software.	Mann-Whitney U test, Student’s *t*-test, Welch’s F test, ROC curve analysis, PLS-DA, Shapiro-Wilk test	1-methylhistidine, 3-methylhistidine, Alanine, arginine, argininosuccinic acid, asparagine, aspartic acid, citrulline, carnosine, 39 metabolites	In PCa samples, taurine was present at significant higher level, while γ-amino- The PLS-DA model built on selected metabolites achieved sensitivity and specificity of 89.47% and 73.33%, respectively, whereas the total group membership classification value was 82.35%.
[40]	Aggio et al. (2015)	73 with haematuria and poor stream without cancer/58 PCa; 24 BC	Urine collection: before PBs Storage temperature: −20 °C Headspace creation: samples were defrosted in water bath at 60 °C for 30 s, mixed with 0.75 mL of 1 M sodium hydroxide, reimmersed in water bath at 60 °C for 50 min EN analysis: 2 cm^3^ of headspace were extracted and analysed	Hybrid GC-MOS sensor system: It is composed of a gas chromatography (GC) oven fitted with a commercially available capillary column interfaced to a heated (450 °C) metal oxide sensor (MOS chemresistor composite of tin oxide and zinc oxide 50:50 by wt). The injection port of the GC was fitted with a 1 mm quartz linear and heated to 150 °C. Cylinder air at 35 psi was used as carrier gas. The temperature program was: 30 °C held for 6 min, up to 100 °C at 5 °C/min, hold 100 °C for 22 min. Volatile organic compounds (VOCs) exiting the GC column reach the MOS sensor, which resistance was recorded.	LOOCV; DoubleCV; SVM-P; Monte Carlo permutation	none	LOOCV: sensitivity 95%, specificity 96%; DoubleCV: sensitivity 87%, specificity 99%; SVM-P: sensitivity with respect to BC 78%; Monte Carlo permutation: chance-like accuracy 50%

**Table 4:** Concentrations of proposed biomarkers in PCa samples with respect to controls.

Biomarkers Proposed	Concentrations in PCa Samples with Respect Controls	
	Increasing Trend	Decreasing Trend
Sarcosine	[25,28,29,30,31]	[36,38]
Isoleucine	[33]	[32,39]
Threonine	[33]	[32,39]
Proline	[31,33,36,37,38]	-
Citrulline	[31,33,36,37,38]	-
Homocitrulline	[31,33,36,37,38]	-
Histidine	-	[37,39]
Methylhistidine	-	[37,39]
Serine	-	[32,39]
Methionine	[33]	[39]
Tyrosine	[33]	[32,37,39]
Arginine	[36]	[39]
kynurenic acid	[27]	[38]
Uracil	[27,38]	-
Glutamine	-	[32,39]

**Table 5:** Advantages and disadvantages of different approaches reviewed.

Approaches Considered	Pros	Cons
**Trained dogs**	Highest diagnostic accuracy achieved	Influence of the discriminative ability on experimental protocol adopted; expensive and time-intensive dog training
**Electronic noses**	Rapid and relative inexpensive analysis	No uniformity concerning sample preparation, analysis and data processing techniques
**Chemical analysis**	Identification and quantification of possible PCa biomarker	Divergent opinions upon the concentrations of same metabolites in PCa samples with respect to controls; time-intensive analysis

## Abbreviations

10FoldCV	repeated 10-fold cross validation
ACN	acetonitrile
AUC	accuracy
BC	bladder cancer
BHP	benign prostate hypertrophy
CI	confidence interval
DDLLME	dispersive derivatisation liquid–liquid microextraction
DMF	dimethylformamide
DoubleCV	double cross validation
DRE	digital rectal examination
EN	electronic nose
ESI	electrospray ionisation
GC-MS	gas chromatography-mass spectrometry
GS	Gleason score
LC-MS	liquid chromatography-mass spectrometry
LDA	linear discriminant analysis
LOOCV	leave one out cross validation
MAD	microwave assisted derivatisation
MOS	metal oxide semiconductors
PBs	transrectal ultrasound-guided prostate biopsy
PCa	prostate cancer
PCA	principal component analysis
PLS-DA	partial least squares discriminant analysis
PSA	prostate specific antigen
Put	putrescine
RF	random forest
ROC	receiver operating characteristics
Spd	spermidine
Spm	spermine
SPME	solid phase microextraction
SVM	support vector machine
SVM-P	Support vector machine-polynomial
UPLC	ultra-high performance liquid chromatography
VOCs	volatile organic compounds
